# Is TLE1 Expression Limited to Synovial Sarcoma? Our Experience at Shifa International Hospital, Pakistan

**DOI:** 10.7759/cureus.6259

**Published:** 2019-11-29

**Authors:** Zafar Ali, Asna Haroon Khan, Usama Rehman, Muhammad Faisal, Imran N Ahmad, Nadira Mamoon, Humaira Nasir, Zujajah Hameed

**Affiliations:** 1 Histopathology, Shifa International Hospital, Islamabad, PAK; 2 Pathology, Shifa International Hospital, Islamabad, PAK; 3 Histopathology, Pak Red Crescent Medical and Dental College, Lahore, PAK

**Keywords:** synovial sarcoma, tle1

## Abstract

Introduction: Synovial sarcoma (SS) accounts for 10-15 percent of adult soft tissue sarcomas. Transducin-like enhancer of split 1 (TLE1), a transcriptional repressor, is essential in hematopoiesis, neuronal differentiation, and terminal epithelial differentiation. TLE1 proteins inhibit Wnt signaling and other cell fate determination signals, and so have an established role in repressing differentiation. TLE1 has recently been shown to be a highly sensitive and relatively specific marker of SS.

Materials and methods: Study design is retrospective, descriptive. A total of 25 cases of SS and 28 of soft tissue lesions were retrieved from the record. TLE1 (clone 1F5) expression was evaluated and scored as negative (<5% of cells positive), 1+ (5-25% of cells positive), 2+ (26-50% of cells positive), or 3+ (>50 % of cells positive).

Result: Twenty-four out of twenty-five (96%) cases of SS showed 3+ TLE1 expression. One (4%) case of poorly differentiated SS showed 2+ positivity. 3+ TLE1 positivity was seen in one (100%) case each of infantile fibrosarcoma and low-grade fibromyxoid sarcoma, while two cases (100%) of schwannoma also showed 3+ positivity. All cases of solitary fibrous tumor) (n=2), clear cell sarcoma of tendons and aponeurosis (n=2), embryonal rhabdomyosarcoma (n=1), and de-differentiated liposarcoma (n=2) showed 2+ positivity. 1+ positivity was seen in alveolar soft part sarcoma (n=2), Ewing's sarcoma (n=4), undifferentiated pleomorphic sarcoma (n=1), myxoid liposarcoma (n=1) and malignant peripheral nerve sheath tumor (n=1). TLE1 was negative in all cases of chordomas (n=2), lipomas (n=2), nodular fasciitis (n=2), malignant perivascular epithelioid cell tumor (n=1) and dermatofibrosarcoma protuberans (n=1).

Conclusion: TLE1 may be a reliable immunostain for diagnosing SS, but its expression is not limited to SS. Its expression should be interpreted in the light of morphological features and a panel of antibodies.

## Introduction

Synovial sarcomas (SS) are aggressive soft tissue tumors with relatively high rates of recurrences and metastases. It classically occurs in the extremities of young adults. Young adult males are commonly affected. The knee joint is the most common site of involvement. SS is a distinct entity that is morphologically and genetically defined. Therefore, reliable diagnostic measures are inevitable [1]. Morphologically it can show various patterns, i.e., biphasic, monophasic, poorly differentiated, and calcifying. Owing to SS heterogeneity, a broad morphological differential creates a diagnostic challenge. Accurate diagnosis is important as SS is responsive to chemotherapy when compared with other soft tissue sarcomas. SS has highly specific genetic features, i.e., SYT-SSX fusion, as a result of t(X; 18) translocation, wherein SSX gene on chromosome X fuses to SYT gene on chromosome 18 [2]. Numerous immunohistochemical (IHC) markers are currently available, including epithelial membrane antigen (EMA), cytokeratin (CK) cocktails, i.e., CK7, CK19, BCL-2, CD99, CD56. A combination of EMA and CD99 is thought to be specific for SS in an appropriate setting. However, this is far difficult to practice in real life, as CD99 is positive in various tumors.

Transducin-like enhancer of split 1 (TLE1) is a transcriptional corepressor of Wnt signaling pathway. TLE1 is one of the transducer-like enhancer genes involved in hemopoiesis, neuronal, and terminal epithelial differentiation. TLE1 has been shown to be a highly sensitive and relatively specific immunohistochemical (IHC) marker of SS [3]. Its diagnostic utility is higher when molecular testing is not available. TLE1 expresses in other soft tumors as well; a recent study showed TLE1 expression in 82% of schwannomas, 39% in rhabdomyosarcoma, and 30% of malignant peripheral nerve sheath tumors [4]. Hence, there is a question on the reliability of TLE1 as solely diagnostic for synovial sarcoma. We evaluated the frequency and intensity of TLE1 staining in synovial sarcomas and other soft tissue lesions.

## Materials and methods

Study design

A retrospective, descriptive study of records from 2012 to 2018 was performed at the Shifa International Hospital, Islamabad, Pakistan.

Sample size

A total of 25 cases of synovial sarcoma (SS) and 28 cases of other soft tissue lesions on which Transducin-like enhancer of split 1 (TLE1) immunostain was applied were retrieved from the records. 

Sampling technique

Non-probability consecutive sampling technique was used.

Data collection methods

All cases of synovial sarcoma and other soft tissue tumors on which TLE1 immunostain was applied were included in the study.

Data analysis plan

TLE1 immunostain (clone 1F5) slide was evaluated independently by two pathologists and scored (Table 1). The scoring system was adopted from the article by Kosemehmetoglu and colleagues [5].

**Table 1:** TLE1 immunostain expression scoring criteria

Score	Percentage of positive cells
Negative	(<5% of cells positive)
1+	(5-25% of cells positive)
2+	(26-50% of cells positive)
3+	(>50 % of cells positive)

## Results

**Figure 1:**
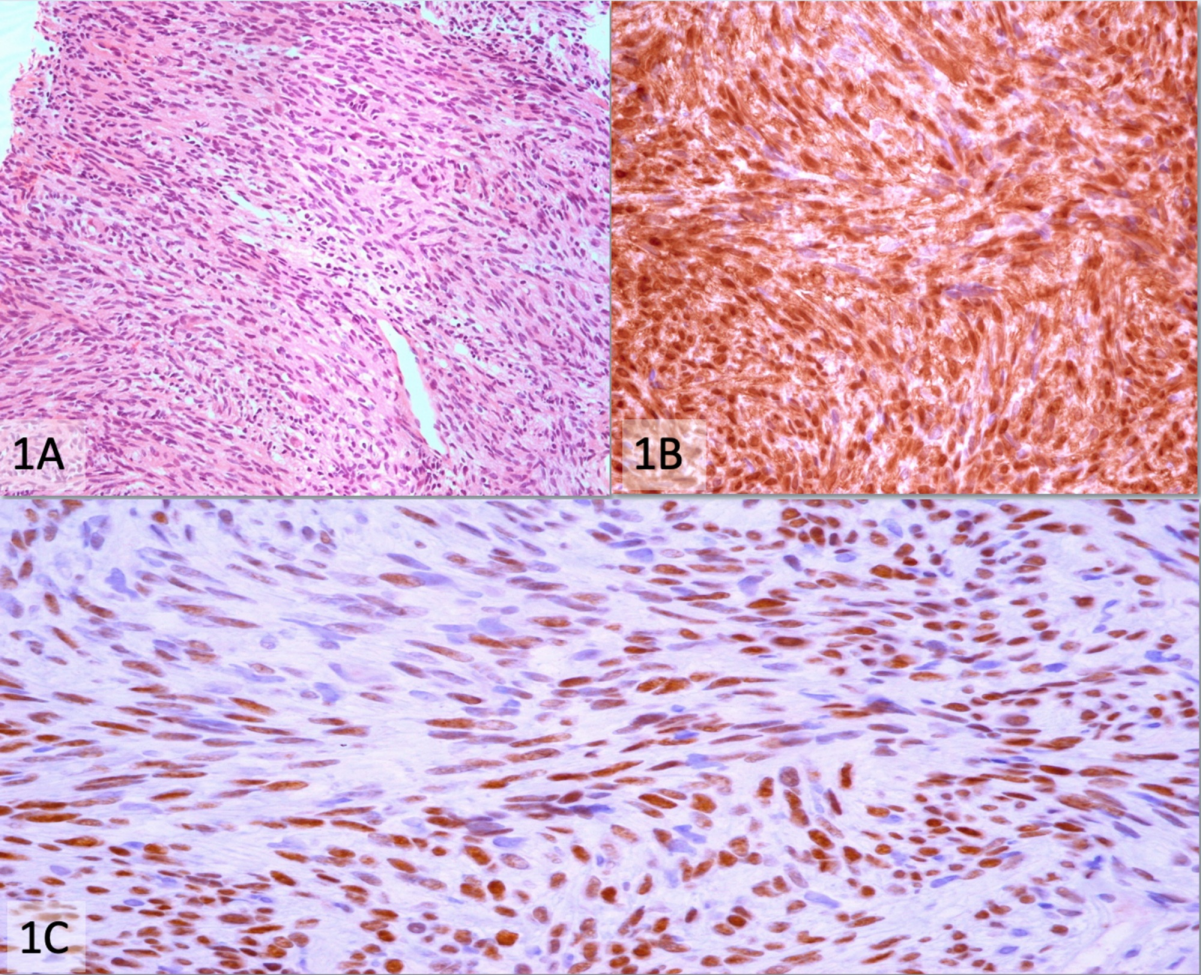
Schwannoma (A) Hematoxylin and eosin stain (200x), (B) Strong and diffuse positivity for S100 immunostain (400x), (C) 3+ positivity for TLE1 immunostain (400x)

Twenty-four out of twenty-five (96%) cases of synovial sarcoma (SS) showed a 3+ Transducin-like enhancer of split 1 (TLE1) expression. One (4%) case of poorly differentiated SS showed 2+ positivity. 3+ TLE1 positivity was seen in one (100%) case each of infantile fibrosarcoma and low-grade fibromyxoid sarcoma, while two cases (100%) of Schwannoma also showed 3+ positivity (Figure 1).

All cases of solitary fibrous tumors (n=2), clear cell sarcoma of tendons and aponeurosis (n=2), embryonal rhabdomyosarcoma (n=1), and de-differentiated liposarcoma (n=2) showed 2+ positivity. 1+ positivity was seen in alveolar soft part sarcoma (n=2), Ewing's sarcoma (n=4), undifferentiated pleomorphic sarcoma (n=1), myxoid liposarcoma (n=1), and malignant peripheral nerve sheath tumor (n=1). TLE1 was negative in all cases of chordomas (n=2), lipomas (n=2), nodular fasciitis (n=2), malignant perivascular epithelioid cell tumor (n=1), and dermatofibrosarcoma protuberans (n=1) (Table 2).

**Table 2:** Grades of TLE1 expression in various soft tissue tumors

Grades	Soft tissue lesions
3+	Synovial sarcoma (n=23)
	Schwannoma (n=2)
	Low grade fibromyxoid sarcoma (n=1)
	Infantile fibrosarcoma (n=1)
2+	Embryonal rhabdomyosarcoma (n=1)
	Solitary fibrous tumor (n=2)
	De-differentiated liposarcoma (n=2)
	Malignant peripheral nerve sheath tumor (n=1)
	Clear cell sarcoma of tendons and aponeurosis (n=2)
1+	Ewing's sarcoma (n=4)
	Alveolar soft part sarcoma (n=2)
	Myxoid liposarcoma (n=1)
	Nodular fasciitis (n=1)
	Undifferentiated pleomorphic sarcoma (n=1)
0	Lipomas (n=2)
	Chordomas (n=2)
	Dermatofibrosarcoma protuberans (n=1)
	Malignant perivascular epithelioid cell tumor (n=1)

## Discussion

In our study, we found Transducin-like enhancer of split 1 (TLE1) immunostain as a highly sensitive but not a specific marker for synovial sarcoma (SS). We found TLE1 expression in other soft tissue tumors that may enter the differential diagnosis of SS. According to the study by Kosemehmetoglu and colleagues, TLE1 expression was seen in 53 of 143 (37%) non-synovial sarcoma, with 36 such cases (25%) showing 2-3+ positivity [5]. Morphology, immunohistochemistry (IHC) and molecular/cytogenetic confirmation of synovial sarcoma-associated fusion genes remain the ‘gold standard’ for this diagnosis. Molecular testing is not available readily in our country, and it remains a challenge to diagnose difficult cases only based on morphology and IHC.

Foo et al. revealed TLE1 as a sensitive and specific marker for synovial sarcoma that could be helpful to distinguish it from histologic mimics, particularly if moderate or strong staining is observed [6]. In their study, only a small subset of malignant peripheral nerve sheath tumor (MPNST) and solitary fibrous tumor (SFT) showed limited staining for TLE1. A study from India in 2012 established TLE1 sensitivity for the diagnosis of synovial sarcomas to be 95.2% [7]. Its overall specificity was 63.7%, whereas with regards to tumors forming its closest differential diagnoses, its specificity was 72%. In the study, TLE1 immunostaining was positive in 40 of 42 synovial sarcomas (95.2%), with 30 tumors displaying 3+ staining, ten displaying 2+ staining, and two displaying 1+ staining [7]. This was comparable to our results (24/25 TLE1 positive).

Zaccarini et al. evaluated TLE1 and CD99 expression in various carcinomas and evaluated the expression of the SS18 (SYT) gene rearrangement in tumors with TLE1 and/or CD99 expression in 100 various carcinomas [8]. Seven of the 98 cases (7%) of carcinomas showed TLE1 expression, highlighting a potential pitfall in the IHC interpretation for diagnosis of synovial sarcoma, thus making a combination of morphology and immunohistochemistry mandatory for diagnosis [8]. Another study carried out on 26 genetically confirmed cases in the largest institution of Southwest China, showed diffuse immunostaining for TLE1, BCL-2, and CD99 in 91.7%, 95.7%, and 56.0% of the tumors, respectively [9]. This again conforms to our study results of diffuse TLE1 positivity.

## Conclusions

Transducin-like enhancer of split 1 (TLE1) may be a reliable immunostain for diagnosing synovial sarcoma (SS) due to its sensitivity, but its expression is not limited to SS as its presence in a number of soft tissue tumors has been established. TLE1 may be of value in the differential diagnosis of synovial sarcoma and should always be used only in the context of a panel of antibodies. Morphology and immunohistochemistry, in combination with molecular confirmation of synovial sarcoma-associated fusion genes, should remain the 'gold standard' for its diagnosis.
